# UNCOVERING PET OWNERSHIP BENEFITS, CHALLENGES, AND RESOURCES: A SURVEY OF PROFESSIONALS WORKING WITH OLDER ADULTS

**DOI:** 10.1093/geroni/igac059.099

**Published:** 2022-12-20

**Authors:** Jessica Bibbo, Justin Johnson, Jennifer Drost, Margaret Sanders

**Affiliations:** Benjamin Rose Institute on Aging, Cleveland, Ohio, United States; Benjamin Rose Institute on Aging, Cleveland, Ohio, United States; Geriatric Research Summa Health, Akron, Ohio, United States; Northeast Ohio Medical University, Rootstown, Ohio, United States

## Abstract

The human-animal bond can play a vital role in the lives of older adults, including those living with dementia. This study used the stress process model to identify the benefits, challenges, and resources associated with pet ownership professionals had encountered in their work with older adults (OA), persons living with dementia (PWD), and care partners (i.e., caregivers, CP). An interdisciplinary (e.g., social services, healthcare) sample (N=360, 89.97% female, Mage=53.22, SDage=11.84) completed an on-line survey addressing pet ownership issues encountered in their work. A conventional content analysis was conducted to analyze the open-ended items asking the benefits and challenges they had encountered, as well as whether and if they had seen pets shape the relationships between OA/PWD and CPs. The final open-ended item asked for any other experiences or thoughts they would like to share on the issue of pet ownership in older adulthood. Preliminary analyses indicated the central benefits of pet ownership for OA and PWD was companionship. Common challenges were providing basic pet care (e.g., feeding, managing waste) and the financial aspect of ownership. Results indicated that family/caregiver assistance was necessary to keep a pet in an OA/PWD’s home and pets could be a source of caregiver burden. Pets provided OA a sense of purpose. Caring for the pet and the bond shared with the pet were also important resources for OA. Pets provided a focus of attention and were a source of engagement for PWD. Pets were a resource for the professionals to build rapport with their clients.

